# Supplementary material for: Formalized aspect-oriented misuse case for specifying crosscutting security threats and mitigations
Source: PLoS One. 2025 Sep 12;20(9):e0322664. doi: 10.1371/journal.pone.0322664 (PMC12431249; doi:10.1371/journal.pone.0322664)
Supplement: S1 Appendix — (PDF) [file pone.0322664.s001.pdf]

Quiz Questionnaire

Instruction:

1. Please answer all questions, which are organized according to each aspect-oriented misuse case and aspect-oriented mitigations.
2. For each multiple-choice question please:
   - Select only one choice for each question, except those indicated with three asterisks (***)
   - Please **circle** your answer.
   - Justify your choice by further answering the question: “how you acquired the information”.
     - Please clearly indicate the relationship between aspect misuse case and use case. Explain how you referenced both the aspect misuse case specification and use case specification to answer the multiple-choice questions.
     - Please clearly indicate the relationship between aspect mitigation and misuse cases. Explain how you referenced both the aspect mitigation specification and the misuse case specification to answer the multiple-choice questions.
3. A special question is asked for each aspect misuse case and aspect mitigation use case. The following is the question:

   Did you identify any place(s) in the “Threaten Pointcut” / “Mitigate Pointcut” specification, which cause any confusion for you to understand the relationship between *aspect misuse case specification and use case specification / aspect mitigation specification and misuse case specification,* for example, in terms of selecting pointcuts, join point, advice, introduction. **(YES or NO)**
   If your answer is “YES” please list the places which causes confusion and provides a brief explanation

   If you identify any confusion about the relationship between *aspect misuse case specification and use case specification / aspect mitigation specification and misuse case specification,* please **circle** “YES” and describe the confusion.
4. Please make sure your handwriting is recognizable.

1. Aspect Misuse case “Get Unauthorized Access via SQLI.”

A. Which of the following threat pointcut expression is correct in terms of use case “log in”?

1. **POINTCUT** login **PRECONDITION** customer is a registered user **JOINPOINT** 2
2. **POINTCUT** login **BF** customer enter data in the system **JOINPOINT** 1 **&&** 2
3. **POINTCUT** login **BF** system VALIDATES THAT **JOINPOINT** 3
4. **POINTCUT** login **POSTCONDITION** customer login **JOINPOINT** 1

How you acquired the information.

B. Read the given pointcuts carefully.

1. **POINTCUT** register customer **BF** customer enters data into field **JOINPOINT** 1-3 **&&** 5 **&&** 7
2. **POINTCUT** login **BF** customer enters data into field **JOINPOINT** 1 **&&** 2
3. **POINTCUT** transfer money to back account **BF** customer enters data into field **JOINPOINT** 2 **&&** 3 **&&** 5

Which of the following ADVICE expressions is correct in terms of the above given pointcuts?

1. **ADVICE** BEFORE **POINTCUT** ALL **EXCUTE** BTF

2. **ADVICE** AFTER **POINTCUT** 2 && 3 **EXECUTE** BTF
3. **ADVICE** AROUND **POINTCUT** ALL **EXCUTE** BTF
4. **ADVICE** AROUND **POINTCUT** 1 && 2 && 3 **EXECUTE** BTF

How you acquired the information.

C. Did you identify any place(s) in the “Threaten Pointcut” specification, which cause any confusion for you to understand the relationship between aspect misuse case specification and use case specification, for example, in terms of selecting pointcuts, join point, advice, introduction. YES / NO

If your answer is “YES” please list the places which causes confusion and provides a brief explanation

2. Aspect Misuse case “Intercept Communication via SSL Stripping.”

A. Which of the following threat pointcut expression is/ are correct (***)

1. **POINTCUT** transfer money to bank account **PRECONDITION** customer initiates the url **JOINPOINT** 1
2. **POINTCUT** transfer money to bank account **POSTCONDITION** record stored in database **||** record updated in database **JOINPOINT** 2 **&&** 3
3. **POINTCUT** register customer **PRECONDITION** customer initiates the url **JOINPOINT** 1
4. **POINTCUT** login **BF** customer initiates the url **JOINPOINT** 1

How you acquired the information.

B. Read the given pointcuts carefully.

1. **POINTCUT** register customer **PRECONDITION** customer initiates the url **JOINPOINT** 1
2. **POINTCUT** login **PRECONDITION** customer initiates url **JOINPOINT** 1
3. **POINTCUT** transfer money to back account **PRECONDITION** customer initiates url [illegible]

Which of the following ADVICE expressions is correct in terms of the above given pointcuts?

1. **ADVICE** BEFORE **POINTCUT** ALL **EXCUTE** BTF
2. **ADVICE** BEFORE **POINTCUT** 1 **&&** 3 **EXECUTE** BTF
3. **ADVICE** AROUND **POINTCUT** ALL **EXCUTE** BTF
4. **ADVICE** AFTER **POINTCUT** 1 **&&** 2 **&&** 3 **EXECUTE** BTF

How you acquired the information.

C. Did you identify any place(s) in the “Threaten Pointcut” specification, which cause any confusion for you to understand the relationship between aspect misuse case specification and use case specification, for example, in terms of selecting pointcuts, join point, advice, introduction. YES / NO

If your answer is “YES” please list the places which causes confusion and provides a brief explanation

3. Aspect Misuse case “Modify Persistent Data via IDOR.”

A. Which of the following threat pointcut expression is correct?

1. **POINTCUT** register customer **POSTCONDITION** record stored **||** data updated **JOINPOINT** 2
2. **POINTCUT** login **POSTCONDITION** customer successfully login **JOINPOINT** 1
3. **POINTCUT** transfer money to bank account **BF** system VALIDATES THAT **JOINPOINT** 4 **&&** 6

How you acquired the information.

B. **Read the given pointcuts carefully.**

1. **POINTCUT** register customer **POSTCONDITION** data stored **||** data updated **JOINPOINT** 2
2. **POINTCUT** transfer money to bank account **POSTCONDITION** data stored **||** data updated **JOINPOINT** 2 **&&** 3

Which of the following ADVICE expressions is correct in terms of the above given pointcuts?

1. **ADVICE** BEFORE **POINTCUT** ALL **EXCUTE** BTF
2. **ADVICE** AFTER **POINTCUT** 1 || 2 **EXECUTE** BTF
3. **ADVICE** AFTER **POINTCUT** ALL **EXCUTE** BTF
4. **ADVICE** AROUND **POINTCUT** 1 && 2 **EXECUTE** BTF

How you acquired the information.

C. **Did you identify any place(s) in the "Threaten Pointcut" specification, which cause any confusion for you to understand the relationship between aspect misuse case specification and use case specification, for example, in terms of selecting pointcuts, join point, advice, introduction. YES / NO**

If your answer is "YES" please list the places which causes confusion and provides a brief explanation

4. Aspect Misuse case "Deface Page"

A. **Which of the following threat introduction expression is correct?**

1. **INTRODUCTION** deface page **REPLACE** register customer || login || transfer money to bank account.
2. **INTRODUCTION** deface page **REPLACE** register customer **&&** login **&&** transfer money to bank account.
3. **INTRODUCTION** deface page **ASSOCIATE TO** register customer || login || transfer money to bank account **AS** included use case

How you acquired the information.

B. **Did you identify any place(s) in the "Threaten Pointcut" specification, which cause any confusion for you to understand the relationship between aspect misuse case specification and use case specification, for example, in terms of selecting pointcuts, join point, advice, introduction. YES / NO**

If your answer is "YES" please list the places which causes confusion and provides a brief explanation

5. Aspect Misuse case "create Malicious Ads"

A. **Which of the following threat introduction expression is correct?**

1. **INTRODUCTION** create Malicious Ads **ASSOCIATE TO** register customer || login || transfer money to bank account **AS** included use case
2. **INTRODUCTION** create Malicious Ads **ASSOCIATE TO** register customer || login || transfer money to bank account **AS** extended by use case
3. **INTRODUCTION** create Malicious Ads **ASSOCIATE TO** register customer **&&** login **&&** transfer money to bank account **AS** included use case
4. **INTRODUCTION** create Malicious Ads **REPLACE** register customer **&&** login **&&** transfer money to bank account

How you acquired the information.

B. Did you identify any place(s) in the "Threaten Pointcut" specification, which cause any confusion for you to understand the relationship between aspect misuse case specification and use case specification, for example, in terms of selecting pointcuts, join point, advice, introduction. YES / NO

If your answer is "YES" please list the places which causes confusion and provides a brief explanation
