# Supplementary material for: Formalized aspect-oriented misuse case for specifying crosscutting security threats and mitigations
Source: PLoS One. 2025 Sep 12;20(9):e0322664. doi: 10.1371/journal.pone.0322664 (PMC12431249; doi:10.1371/journal.pone.0322664)
Supplement: Attachment — Submitted filename: PLOS.pdf [file pone.0322664.s004.pdf]

Dear editor in chief

Regarding the paper entitled “Formalized Aspect-oriented Misuse Case for Specifying Crosscutting Security Threats and Mitigations” paper ID : PONE-D-24-39206, the authors should consider the following revisions :

1. There is no need to title each paragraph in the abstract; it is sufficient to write them as regular paragraphs, meaning the labels at the beginning of each paragraph (such as context, problem, etc.) should preferably be removed, and the conclusion paragraph at the end of the abstract should be omitted.
2. Don't use active voice (e.g., 'we', 'I'); use the passive voice instead.
3. “Restricted Misuse Case Modelling (RMCM)”, should be added to the keywords
4. Add a related works section after the introduction section and show the gaps of them that the proposal aimed to solve.
5. Need to consider more modern references for the related works section
6. Figure 1 does not clarify where the trigger (starting point) is and where it ends. This detail should be clarified before discussing the figure below.
7. “Formalizing the Aspect Threats using Restriction Rules” and “Formalizing the Aspect Mitigations using Restriction Rules” .These two paragraphs need to be rephrased more clearly and concisely, and better organized, to make them more understandable when reading.
8. Who is the actor and where is the trigger
9. "**Dependent and independent variables section** “It is better to rephrase this paragraph in a numbered list format for the purpose of organization
10. formula to calculate the error rate for each restriction rule , is it the authors' own innovation or taken from a source? If it is taken from a source, it must be properly cited.
11. What are the potential applications that could benefit from the proposed approach.
12. Add a comparison analysis table between the proposed system and the related works
13. Explain system limitation
14. Add future works or recommendations
