# Supplementary material for: Formalized aspect-oriented misuse case for specifying crosscutting security threats and mitigations
Source: PLoS One. 2025 Sep 12;20(9):e0322664. doi: 10.1371/journal.pone.0322664 (PMC12431249; doi:10.1371/journal.pone.0322664)
Supplement: Attachment — Submitted filename: Response to Reviewers.docx [file pone.0322664.s005.docx]

# PLOS Journal Submissions Rebuttal Letter

Dr. Nadeem

Journal Editor

PLOS ONE

17^th^ February 2025

Dear Dr. Nadeem

Thank you for appreciating our study and inviting us to submit a revised manuscript entitled, “Formalized Aspect-oriented Misuse Case for Specifying Crosscutting Security Threats and Mitigations” to PLOS One. We also appreciate the time and effort you and each of the reviewers have dedicated to providing insightful feedback on ways to strengthen our study. Thus, it is with great pleasure that we resubmit our manuscript for further consideration. We have incorporated changes that reflect the detailed suggestions you have provided. We also hope that our revision and the responses we provide below satisfactorily address all the issues and concerns that have been shared.

To facilitate review of our revisions according to *Comments by Academic Editor* and *Review Comments by Reviewers*, the following is a point-by-point response to the questions and comments delivered in email dated 8 January 2025. Additionally for ease, we also referenced each change in the manuscript “Revised manuscript with track changes” using the Word comment feature, relating it to the corresponding reviewer comment number.

**Comment by academic editor**

- The manuscript must describe a technically sound piece of scientific research with data that supports the conclusions. Experiments must have been conducted rigorously, with appropriate controls, replication, and sample sizes. The conclusions must be drawn appropriately based on the data presented.

**REPONSE:** We appreciate your valuable feedback and understand the importance of addressing the points mentioned in review. We have incorporated the reviewers' feedback into the revised manuscript, which now includes a comprehensive explanation of the concerns raised as justification to sample size, student backgrounds, case study scope, reason of student difficulties with detailed results, detailed literature review with highlighted key differences, need of tool support, scalability, evaluation metrics and others, alongside improvements in Grammer and Spellings. We also added a " Future Work" section that addresses the suggestions given by the reviewers.

- The [PLOS Data policy](http://track.editorialmanager.com/CL0/http:%2F%2Fwww.plosone.org%2Fstatic%2Fpolicies.action%23sharing/1/010f019442dbe301-deaa4d3c-1f8a-45aa-8f02-bf8cf5d21dfc-000000/Cw86V-NuLEOpSD9gkP17Cct8D_cL2Xt5Q9jA3d0avSU=192) requires authors to make all data underlying the findings described in their manuscript fully available without restriction, with rare exception (please refer to the Data Availability Statement in the manuscript PDF file). The data should be provided as part of the manuscript or its supporting information, or deposited to a public repository. For example, in addition to summary statistics, the data points behind means, medians and variance measures should be available. If there are restrictions on publicly sharing data—e.g. participant privacy or use of data from a third party—those must be specified.

**RESPONSE:** As per your suggestion, we have ensured full accessibility to the data without any restrictions. The complete dataset is provided as supporting files in archived folder titled “collected data for each metric” containing one file, titled "Error Rate Calculation," includes detailed data gathered to calculate the error rate for each rule by each subject, covering the AFMUC approach comprehensively. Another file, titled "Ambiguity-Understandability, Applicability, Restrictiveness, and Error Rate," contains aggregate data measuring the ambiguity of AFMUC in terms of understandability, applicability, restrictiveness and error rate. We have also provided “statistical test data” and the results analyzing the relationship between the measures of ambiguity as a supporting file. The data was collected anonymously, adhering to all necessary ethical approval procedures. To promote transparency, the entire dataset has been made fully available.

- PLOS ONE does not copyedit accepted manuscripts, so the language in submitted articles must be clear, correct, and unambiguous. Any typographical or grammatical errors should be corrected at revision, so please note any specific errors here.

**RESPONSE:** We have carefully revised the manuscript, addressing all typographical and grammatical errors. The text has been carefully proofread and subjected to a comprehensive grammar check to ensure clarity, accuracy, and readability throughout the manuscript.

**Comments by reviewers**

**REVIEWER 1 COMMENTS:**

1. As the authors state, the results are limited to one set of 24 students. I would have preferred seeing the results over multiple cohorts of students.

**RESPONSE:** We accept that the results are currently limited to one cohort of 24 students. primarily because of ease of access to students with the required technical skill set. As per your comments, we have revised the manuscript with comprehensive justification in section “population and sampling” with detailed justification of our population also we have updated the section “conclusion validity” to address this potential threat to study.

2. The students don't have the ideal background for the case study, and other than possibly having some courses in common, may have significantly different backgrounds.

**RESPONSE:** We have selected the students based on defined criteria for technical skills set in terms of the courses studied. Additionally, to ensure their consistent background and their preparedness before the experiment, we gave them the training session on AFMUC and its fundamental concepts. Moreover, the training session included hand on experience to practice the AFMUC, and graded assignment was designed to assure the shared understanding of the concepts before the experiment. These measures were implemented to idealize students’ knowledge and skills according to the required criteria. As per your comments, we have now comprehensively detailed these efforts in the manuscript, under the section “Population and Sampling Strategy” and “Training of subject” section with the detailed background courses and training procedure respectively.

3. The case study is very small. A single application of the method in a small case study of 3 hours is not really adequate to determine the efficacy of the method.

**RESPONSE:** We agree with your comment. The case study is small, with a duration of only three hours. The size of case study was kept small intentionally because our objective was to fully demonstrate the proposed AFMUC approach, instead of covering all aspects of case study. The rationale for the size of case study and timeframe of the experiment has been outlined and revised in the manuscript under the headings “Case study: mobile finance application” to justify the selection of case study. "Construct Validity" to justify the size of the case study and "External Validity” to justify the selection of time.

4. I did not see adequate support for the conclusion that students faced difficulties, in some cases, due to lack of exposure to aspect-oriented modelling. The evidence for this conclusion and more generally for the usefulness of the method/template is lacking.

**RESPONSE:** We have observed the difficulty of students based on analysis of quantitative data under the measure of error rate. The high error rate while applying the essential concepts of the approach as pointcut, joinpoint expression and advice type shows that students face difficulty while applying those in practical scenario. which we believe were partly due to their limited exposure to aspect-oriented modeling (AOM). Based on comments, we comprehensively addressed this and revised the section “error Rate” with detailed analysis of results in line with this comment.

5. On page 3 under item 4, you propose extensive training, but it's unknown whether that would help. More work is needed to make this determination.

**RESPONSE:** We appreciate your insightful suggestions. We agree that providing more details about the extensive training and results of the study would enhance the clarity of our work. In the revised work, on page 3 under the "Introduction" section we have clarified this point and provided a more comprehensive overview. We hope this revision better conveys the depth of the training and the context of the study results.

6. I would suggest that the authors repeat the experiment with a different group of students, perhaps at another university and with more consistent backgrounds, perform the extensive training to remove ambiguity in the results, and use something other than opinion surveys to get a more objective assessment of the results. I also recommend replicating the experiment with an industry partner to get a different audience and compare those results to the student results.

**RESPONSE:** We appreciate your comments and suggestions. We also believe that our research study has opened possibilities for other researchers to replicate the experiment or perhaps perform a larger experiment with more comprehensive results. We acknowledge the importance of these suggestions and revised the manuscript while incorporates these as open research directions in the newly added section “Future work”

7. There are many places where the English usage is awkward, and for unknown reasons, typos have been introduced. I don't know if the authors wrote the paper in another language and then attempted to translate it into English, OR if they attempted to write it in English and didn't proofread it adequately. If accepted for publication, these problems must be fixed. Some examples follow: Under the results summary on the first page "rather face difficulty" should be "rather than face difficulty". On page 2, "lack to", where it should be "fail to", also, "excessive" should be "extensive". On page 5, replace "flow is known" with "flow and is known". On other pages, "INTRODUCTION" is misspelled as "INTROCUDTION" and "EXECUTION" is misspelled as "EXCUTION". These are just examples, I did not do a thorough edit, since this is what the authors need to do.

**RESPONSE:** We appreciate your careful review and understand the importance of clear and accurate language in conveying our work. We thoroughly proofread the manuscript. On the first page the result summary is revised. and correct all identified issues, including the examples you mentioned, to ensure the language is smooth and the manuscript is ready for publication

**REVIEWER 2 COMMENTS:**

1. Literature Review Depth:
   While the paper briefly touches on related work, it could benefit from a deeper exploration of existing methodologies for modeling security threats. Highlighting key differences would strengthen the argument for the proposed approach.

**RESPONSE:** In response to your feedback, we have updated the literature review to provide a more in-depth exploration of existing methodologies for modeling security threats. We update literature studies with more details. Each sub section under related work is revised with the key differences and at the end, we also give a research gap which strengthens the need of our approach. We have revised the “related work” section with details and added new subsection as “Research gap analysis”

2. Consider incorporating a case study or an application to a real-world system. This would provide tangible evidence of the method's effectiveness and practicality.

**RESPONSE:** We have considered incorporating the case study to evaluate the effectiveness and applicability of the proposed AFMUC approach. We have used the Mobile financing case study (MFA) obtained from the industry partner. To maintain the confidentiality agreement, we anonymized it as company “X”. We have obtained the software requirements specification of MFA including security requirements. With the help of security experts from industry and two experts from academia from the information security domain, we have designed threats and mitigations for MFA to prepare the case study in the context of this research.

3. It would be helpful to discuss or propose tool support for implementing the formalized aspect-oriented misuse cases. This addition could significantly enhance adoption by practitioners.

**RESPONSE:** We agree that discussing tool support for implementing formalized aspect-oriented misuse cases (AFMUC) would greatly enhance the adoption of the method by practitioners. We have discussed the need of tool support in sub section “error rate” under the main section “Results and Analysis” Additionally, we have provided this suggestion as open direction in the future work sections of the manuscript.

4. The evaluation of the approach lacks quantitative metrics. Including metrics or benchmarks for comparison would lend credibility to the claims of effectiveness and efficiency.

**RESPONSE:** We have included the metrics to claim the effectiveness and efficiency of the proposed approach for proposed study. Since AFMUC is a novel approach, without having established benchmarks for comparison, we have defined quantitative metrics to evaluate the understandability, applicability, restrictiveness, and error rate for the AFMUC approach. We have designed the questionnaire to collect quantitative data for these metrics and design formulas to calculate the error rate for each AFMUC rule. The measures for these metrices are adopted from the literature and referenced in the manuscript. For evidence we also have provided the supporting files (collected data for each metric) representing the data for these metrices i.e., understandability, applicability, restrictiveness, and error rate with the revised submission. Additionally, the results and analysis section included the findings for each metric—understandability, applicability, restrictiveness, and error rate—along with their statistical analysis. To address your comments clearly in the manuscript, we have revised the manuscript by adding a new point as “data collection” under the main section “Experimentation”

5. The methodology’s scalability for large-scale systems remains unclear. Expanding on this aspect or conducting experiments on larger systems would strengthen the paper.

**RESPONSE:** We appreciate your suggestions. The proposed approach is scalable to a larger context. To address this, we have expanded this aspect in the discussion section of the manuscript. Also to further evaluate the scalability of the approach, we provided open directions in the section “Future Work”.

6. Clarity in Terminology:
   Some terms, such as "crosscutting concerns," might not be familiar to all readers. A brief explanation or glossary would aid understanding.

**RESPONSE:** Thank you for your thoughtful suggestion. We agree that terms like "crosscutting concerns" may not be familiar to all readers. To address this, we have added a brief explanation of this term in the "Introduction" section.

7. Grammar and Syntax:
   A thorough proofreading is recommended to eliminate minor grammatical errors and ensure smooth readability.

**RESPONSE:** Thank you for your feedback. We are sorry for such mistakes. We have thoroughly proofread the manuscript to eliminate any minor grammatical errors and ensure smooth readability.

8. Visual Aids:
   While the diagrams are helpful, improving their clarity (e.g., using color coding or annotations) could enhance their effectiveness.

**RESPONSE:** Thank you for your suggestion. We make these adjustments according to the PLOS figures guidelines in the revised version to ensure the diagrams are more visually clear and informative. We also gave the color codes to the results in graphical charts under the section results and analysis for understandability, applicability, restrictiveness and error rate.

9. Consistency in Formatting:
   Ensure consistency in the formatting of headings, citations, and references for a professional presentation.

**RESPONSE:** Thank you for your feedback. We have followed the PLOS ONE style guidelines for formatting headings, citations, and references to ensure consistency and maintain a professional presentation. We carefully review these elements to ensure full adherence to the guidelines in the revised version.

10. Future Work Section:
    Expanding the future work section to outline specific research directions or challenges would provide valuable insights for readers interested in this field.

**RESPONSE:** Thank you for your suggestion. We have added a "Future Work" section to the manuscript, where we outline specific research directions and challenges. We believe this addition will provide valuable insights for readers interested in further exploring this study.

**CONCLUDING REMARKS**: Again, thank you for giving us the opportunity to strengthen our manuscript with your valuable comments and suggestions. We have worked hard to incorporate your feedback and hope that this revision convinces you to accept our submission.

Sincerely,

Shumaila Iqbal

Corresponding Author

Riphah International University

Department of Computing

Islamabad

Shumaila.iqbal@riphah.edu.pk
