# Supplementary material for: Formalized aspect-oriented misuse case for specifying crosscutting security threats and mitigations
Source: PLoS One. 2025 Sep 12;20(9):e0322664. doi: 10.1371/journal.pone.0322664 (PMC12431249; doi:10.1371/journal.pone.0322664)
Supplement: Attachment — Submitted filename: Response to Reviewers updated.docx [file pone.0322664.s006.docx]

# PLOS Journal Submissions Rebuttal Letter

Journal Editor

PLOS ONE

Date 28^th^ May 2025

Dear Dr. Jason Morgan

Thank you for appreciating our study and inviting us to submit a revised manuscript entitled, “Formalized Aspect-oriented Misuse Case for Specifying Crosscutting Security Threats and Mitigations” to PLOS One. We appreciate the reviewers’ continued efforts in evaluating our manuscript and their insightful comments in this second round of review. We have carefully considered each point raised and have revised the manuscript accordingly to address the remaining concerns. In the following responses, we provide a detailed account of the changes made and the rationale behind them. We believe that these revisions have strengthened the quality and clarity of the manuscript, and we hope that it now meets the standards for publication.

To facilitate review of our revisions according to *Comments by Academic Editor* and *Review Comments by Reviewers*, and attached PDF file, the following is a point-by-point response to the questions and comments delivered in email dated 30^th^ April 2025. Additionally for ease, we also referenced each change in the manuscript “Revised manuscript with track changes” using the Word comment feature, relating it to the corresponding reviewer comment number.

**Comments from PLOS Editorial Office:**

We note that one or more reviewers has recommended that you cite specific previously published works in the current and previous rounds of revision. As always, we recommend that you please review and evaluate the requested works to determine whether they are relevant and should be cited. It is not a requirement to cite these works and you may remove any added citations before the manuscript proceeds to publication. We appreciate your attention to this request.

**Response:**

We carefully reviewed the comment regarding citation of previously published works recommended by the reviewers in the current and earlier revisions. In the current revision, Reviewer #3 suggested citing the following work: [*https://link.springer.com/chapter/10.1007/978-3-031-58388-9_8*](https://link.springer.com/chapter/10.1007/978-3-031-58388-9_8)*.*

After thoroughly reviewing this suggested paper, we determined that it does not directly align with our study's primary objective or in related work section, which is regarding the modeling crosscutting security threats and corresponding mitigations. However, it does effectively emphasize the broader importance of addressing security in software applications.

In recognition of this relevance, we have incorporated this citation into the “**Introduction**” section to contextualize the significance of security requirements in modern software applications. We believe this addition appropriately acknowledges the contribution of the suggested work without misrepresenting its scope.

Additionally, in response to reviewer’s comments, we also have revised our references across the manuscript to enhance current and more relevant existing work. A detailed breakdown of these changes, along with the justification including comment number and corresponding citations, is provided below for clarity and ease of review.

| **New reference/citation added in manuscript** | **Year** | **Justification + comment #** | **Changes made in manuscript** |
| --- | --- | --- | --- |
| [1] | 2024 | Added to enhance the need for security in software applications.  Suggested by reviewer#3 comment # 3 | Introduction section |
| [4] [5] [6] | 2024, 2021, 2019 respectively | Added to justify the need of misuse cases and they are simple and easy as compare to other threat modelling approaches.  Reviewer 2 comment 3 and 5 | Introduction section |
| [12][13] | 2022, 2014 respectively | Added AOM based OR misuse case based existing approaches, add recent work  Reviewer 2 comment 9,11 Attached PDF comment 5, 12 | Related work |
| [42] [43] [44] | 2021, 2022, 1999 | Comparison to existing threat modelling approaches STRIDE DREAD etc.  Reviewer 2 comment 5, attached PDF comment 12 | Discussion-> Comparison to existing work |

**Comments by reviewers**

**REVIEWER 1 COMMENTS:**

1)Although the paper is now easily readable, there are still some places where English phrases appear that are not sentences, generally because a verb is missing.

**Response:**

We appreciate your observation, which has helped us improve the overall clarity and readability of the paper. We thoroughly reviewed the manuscript and carefully revised all sentences where verbs were missing or where phrases did not form complete sentences.

2) While I still would prefer seeing additional experimental results prior to publication, the Future Work section partially addresses this concern.

**Response:**

We sincerely thank the reviewer for this thoughtful and constructive comment. We fully acknowledge the importance of broader empirical evaluation and agree that additional experimental results would further enhance the rigor and applicability of our proposed AFMUC approach. However, given the current scope and objectives of this study, our focus has been on the initial verification and validation of the foundational AFMUC approach.

Based on the reviewer’s comment, we have revised the “**Future Work”** section to explicitly state our plan to extend this work with additional experiments, including larger sample sizes and real time case studies. We are committed to pursuing this direction in the next phase of our research and believe it will meaningfully complement the current findings.

**REVIEWER 2 COMMENTS:**

1. Clarity of Terminology:
   In the abstract and introduction, it would be helpful to provide a brief definition or explanation of aspect-oriented misuse cases for readers who might not be familiar with this specific terminology.
   Consider clarifying the term "crosscutting security threats" early in the paper to ensure it is clearly understood in the context of your framework.

**Response:**

Considering the comment we have updated the “**Abstract**” and “**Introduction**” sections with the brief definition of terminologies aspect-oriented misuse cases and crosscutting security threats. The detailed concepts of the AFMUC approach e.g., aspect-oriented misuse cases and mitigation cases are provided on pages 4 and 5 under the heading *AFMUC Construct*, where we define essential AOM constructs including aspect, pointcut, joinpoint, advice, and introduction core definition and then in the context of proposed approach. Furthermore, this section has been updated to include the definition of *weaving*, clarifying how the weaving process is applied within the context of the proposed AFMUC approach. We acknowledge this omission in the original version and appreciate the opportunity to address it.

2. Introduction Section:
   The introduction provides a good overview of the problem, but it would benefit from a more explicit explanation of how the proposed method improves upon existing approaches. Perhaps adding a sentence comparing it to the state-of-the-art in threat modeling or misuse case analysis could help readers better understand the novelty and importance of your work.

**Response:**

The introduction section has been updated to include a clear statement emphasizing the novelty of the proposed AFMUC approach. A comparative sentence has been added to highlight how it improves upon existing misuse case analysis methods. This enhancement provides readers with a better understanding of the contribution and relevance of our work in relation to the state-of-the-art misuse case approach.

Additionally, a comprehensive comparison of AFMUC with other state-of-the-art threat modelling approaches and existing misuse case approached has been included in the **Discussion** section. This comparison is structured into three focused sub-parts:

i. *Comparison with existing (mis)use case approaches*,
ii. *Comparison with UML- profile based approaches*, and
iii. *Comparison with traditional threat modeling approaches*.

These comparisons provide a critical evaluation of how AFMUC improves upon and differs from existing approaches, enhancing the clarity and relevance of our contribution. The integration of this content ensures that readers and reviewers can better appreciate the scope, innovation, and impact of our work.

3. Motivation and Justification:
   While the motivations for using aspect-oriented modeling are explained, it could be useful to include a more detailed justification of why misuse cases specifically are an appropriate vehicle for specifying security threats in comparison to other threat modeling techniques.

**Response:**

We have revised the “**Introduction”** section to include a justification for using the misuse case as threat modelling approach to model security threats. Specifically, we now explain the rationale for selecting misuse cases over other threat modeling techniques such as STRIDE, DREAD, and others. The added citation numbers are [4] [5].

To support this revision, we have cited three relevant and recent studies:

- A practitioner survey: *"Security in Agile Software Development: A Practitioner Survey"* (2021), and
- A systematic literature review: *"A Systematic Review and Analytical Evaluation of* Security Requirements Engineering Approaches" (2019).
- Comparative study “A Comparative Analysis of Threat Modelling Methods: STRIDE, DREAD, VAST, PASTA, OCTAVE, and LINDDUN,” (2024)

These studies reinforce the selection of misuse case by demonstrating that it remains one of the most widely adopted techniques by both researchers and practitioners. The cited literature highlights its advantages in simplicity, ease of use, and effectiveness in identifying security threats alongside functional requirements, making it particularly suitable for early analysis of security.

The references have been incorporated into the manuscript, and the relevant sentence in the **“Introduction”** section now explicitly connects this justification to the broader context of threat modelling.

4. Methodology Section:
   The methodology section could be expanded to provide more examples or case studies to illustrate how your approach can be applied to real-world security scenarios.

**Response:**

We appreciate the reviewer’s suggestion to expand the methodology section by providing more examples or case studies to illustrate the practical application of our approach. In the current version of the manuscript, we have presented two case studies to exemplify, verify, and validate the proposed AFMUC approach:

1. **EU-Rent Car Rental System** – a well-established, commonly used academic case study that allows for standardized demonstration of modeling approaches. (exemplify)
2. **Mobile Financing Application** – a real-world industrial project obtained through collaboration with an industry partner, used to assess the practical applicability of our proposed approach. (verification and validation)

To address the reviewer’s comment more explicitly, we have revised the “**Future Work”** section to emphasize the importance of further empirical evaluation. We now include a statement highlighting our intention to apply AFMUC to additional case studies and real-world applications in diverse domains to further validate its scalability, adaptability, and effectiveness in various security-critical applications.

We hope this update sufficiently addresses the reviewer’s concern and demonstrates our commitment to ongoing evaluation of the proposed methodology.

5. It would be useful to include a brief comparison of the proposed approach with traditional threat modeling techniques like STRIDE or DREAD to highlight its unique contributions.

**Response:**

We have expanded the “**Discussion”** section of the manuscript to include a more detailed comparative analysis. Specifically, we introduced three dedicated subheadings:

1. *Comparison with existing (mis)use case approaches*
2. *Comparison with UML- profile based approaches*
3. *Comparison with traditional threat modeling approaches*

Under the third subheading, we present a focused comparison of AFMUC with traditional threat modeling approaches such as STRIDE, DREAD, and Attack Trees. This comparison highlights the unique contributions of AFMUC, particularly its ability to integrate security concerns directly with functional requirements and to support behavioral modeling in a unified manner.

We believe this extended discussion significantly enhances the manuscript by more clearly positioning AFMUC within the broader context of security modeling methods and its added value over traditional threat modelling approaches.

To support the comparison, the added citations are

6. Figures and Diagrams:
   The diagrams illustrating the aspect-oriented misuse cases are very helpful, but some of them might benefit from additional labeling or annotations to make them easier to follow for readers less familiar with this modeling technique.
   In Figure X, it might be helpful to add more context or a legend to explain the relationships between the components, especially for readers unfamiliar with the specifics of aspect-oriented programming or misuse case methodology.

**Response:**

We have carefully reviewed and revised **Figures 1, 6, 7, 8, and 10**, which illustrate the proposed approach and the aspect-oriented misuse cases. To improve comprehension, we have added additional labels, annotations, and visual cues where necessary to clarify the relationships between components. These enhancements are intended to make the diagrams more accessible and informative, particularly for readers who may be less familiar with aspect-oriented programming or misuse case modeling. We believe these improvements significantly contribute to the overall clarity of the manuscript.

7. Results and Discussion: The results presented are interesting, but the paper would benefit from a more detailed discussion on the limitations of the proposed approach. Are there any scenarios where it might not be suitable?

**Response:**

We have added a dedicated subsection titled **"Limitations"** under the “**Discussion”** section of the revised manuscript. This subsection offers a clear and balanced analysis of the potential constraints and scenarios where the proposed AFMUC approach may encounter challenges or may not be suitable.

8. The paper could also discuss how the framework can be adapted or scaled for different types of applications, particularly when dealing with complex or large systems.

**Response:**

We have expanded the “**Discussion**” section of the revised manuscript to explicitly address the scalability and adaptability of the proposed AFMUC approach. This addition outlines how the proposed approach can be adapted to various types of applications, including complex and large-scale systems, and emphasizes its potential to support security modeling across diverse security-critical domains.

9. Related Work:

The related work section is comprehensive, but it would be helpful to briefly mention recent advancements in the integration of security in aspect-oriented programming (AOP) or misuse case approaches, to situate the work in the broader academic landscape.

**Response**

We have updated the “**Related Work”** section to incorporate recent advancement in integration of security in aspect-oriented programming (AOP) with various UML diagrams, including Aspect-Oriented Sequence Diagrams (Mal sequence) and AspectSM. The added citation numbers are [11] [12] [13].

While these works highlight the application of AOP in different modeling contexts, we did not find any recent or single study that specifically addresses the integration of AOP with misuse cases. This gap further reinforces the novelty of our proposed AFMUC approach and justifies the need to explore AOP integration in misuse case modeling.

The following related work has been referenced to demonstrate relevant contributions in the field:

- *“Does aspect-oriented modeling help improve the readability of UML state machines?”* (2014).- [12]
- *“Modeling and verification of authentication threats mitigation in aspect-oriented mal sequence woven model”* (2022)- [13]

To validate this claim, we have already cited [11] a recent systematic mapping study titled:

*“On the Coverage of Aspect-Oriented Methodologies for the Early Phases of the Software Development Life Cycle”*, published in 2022.

It supports our observation and provides a broader overview of current trends and gaps in AOP-based modeling. These additions not only strengthen the foundation of our work but also strengthen the contribution of AFMUC as a novel approach.

10. Conclusion:
    The conclusion is clear but could be slightly expanded to suggest future work or possible directions for improving the framework. What are the next steps for integrating this approach with real-world security applications or tools?

**Response:**

While a detailed “**Future Work”** section has already been provided. However, to satisfy this comment, the “**Conclusion”** section has also been revised to briefly highlight future directions.

11. References:

    Ensure that all references are correctly formatted according to the journal’s guidelines. A minor formatting issue was noted in reference [X].
    Consider adding a few more recent references (2019 and later) that discuss the application of AOP in security modeling to strengthen the paper's current relevance.

**Response:**

All references have been thoroughly reviewed and updated to ensure compliance with the journal’s formatting guidelines. Few reference are reformatted. Also, recent references are also added. The list of newly added references is as follows:

More references are added in 2019 onward. The details are the following:

| **New reference/citation added in manuscript** | **Year** |
| --- | --- |
| [4] [5] [6] | 2024, 2021, 2019 |
| [13] | 2022 |
| [42] [43] | 2021, 2022 |

12. Minor Typographical/Grammar Issues:

    In the sentence "The aspect-oriented misuse case are designed to handle..." the verb "are" should be replaced with "is" to ensure subject-verb agreement.
    A few minor grammatical errors in sections 4.2 and 5.1. I suggest a careful proofreading or using a grammar-checking tool to address these.

**Response:**

we have thoroughly reviewed the manuscript and also a grammar-checking tool is utilized to ensure overall clarity, accuracy, and readability throughout the manuscript. We appreciate the reviewer’s attention to detail, which has contributed to improving the quality of the manuscript.

**REVIEWER 4 COMMENTS:**

1. Abstract needs to be concise, discuss work done, results, relevance

**Response:**

The abstract has been revised to ensure conciseness while effectively summarizing the work done, findings, and the relevance of the study.

2. Discuss tables, figures, formula extensively

**Response:**

We have revised the manuscript to include a more detailed discussion of the figures, tables and Formula. Detailed explanations have been added where necessary to improve the overall presentation and readability.

- Figures 12, 13, 14, and 15 now provide a detailed presentation of the results obtained from applying the AFMUC approach. such as understanding, applicability, restrictiveness and error rate.
- Table 7 statistical test results and Table 8 comparative analysis of results are explained more explicitly, with details that clarifies the implications of the presented data and how it supports the findings of our study .
- Figures 1, 6, 7, 8, and 10 are annotated in detail to explain the concepts directly within the visuals. As a result, the descriptive text is kept concise, relying on the figure annotations to clarify the concepts.
- Newly added Tables 9,10,11 and 12 are also explained with details. Providing a comprehensive comparison between existing approaches and proposed AFMUC approach.
- However, Tables 1–6 are self-explanatory, with each table including a description of all its elements. Re-explaining them in detail in the text would be redundant and may distract the reader from the main discussion.
- The error rate formula (equation 1) used in the evaluation has been extensively explained, including variable descriptions, and interpretation of its results in the context of model validation.

3. Discuss related works <https://link.springer.com/chapter/10.1007/978-3-031-58388-9_8>

**Response:**

The suggested study provides valuable insights and highlights the importance of addressing security in edge intelligence systems that integrate IoT and AI. While it does not directly align with our related work “crosscutting threat and mitigation modelling in misuse cases”. However, it reinforces the broader relevance of modeling security in today's critical, real-time software applications. Recognizing its contextual significance, we have cited this study in the “**Introduction**” section to strengthen the foundational motivation for our proposed work.

**Comments from attached PDF file**

1. There is no need to title each paragraph in the abstract; it is sufficient to write them as regular paragraphs, meaning the labels at the beginning of each paragraph (such as context, problem, etc.) should preferably be removed, and the conclusion paragraph at the end of the abstract should be omitted.

**Response:** .

As noted in first revision, the abstract has been revised accordingly by removing all titles and labels at the beginning of each paragraph. Additionally, the concluding paragraph has been omitted in line with your suggestion. The abstract has also been refined for clarity and precision to better communicate the core contributions of the work.

2. Don't use active voice (e.g., 'we', 'I'); use the passive voice instead.

**Response:**

The manuscript has been thoroughly revised to eliminate the use of active voices, such as "we" and "I". All relevant sentences have been restructured using the passive voice to maintain an objective and formal tone in line with this comment and academic writing standards. For instance, phrases like “We have piloted the experiment” have been changed to “an experiment is piloted”.

3. “Restricted Misuse Case Modelling (RMCM)”, should be added to the keywords

**Response:** '

Restricted Misuse Case Modelling (RMCM)' has been added to the keywords as recommended.

4. Add a related works section after the introduction section and show the gaps of them that the proposal aimed to solve.

**Response:**

The “**related work”** section has been added after the **“introduction”** section. Furthermore, a “Research Gap Analysis” subsection has also been included to clearly identify the gaps existing approaches in relevance to the proposed approach.

5. Need to consider more modern references for the related works section

**Response:**

We have incorporated recent references in the related work section that reflect the latest advancements in modeling, crosscutting security concerns using aspect-oriented approaches across various UML models.

Specifically, we have cited:

- Aspect-Oriented Sequence Diagrams (2022) citated as[13]
- A 2022 systematic literature review addressing the use of aspect-oriented modeling (AOM) for specifying security requirements in UML citied as [11]

These references have been included to reinforce the foundation of our proposed AFMUC approach and to acknowledge relevant efforts in addressing security concerns using AOM within different UML modeling contexts.

6. Figure 1 does not clarify where the trigger (starting point) is and where it ends. This detail should be clarified before discussing the figure below.

**Response:**

The figure has been updated to clearly indicate the trigger (starting point) and the endpoint of the process using labels as “AFMUC development phase” and “AFMUC use phase”. These details have been explicitly labeled to guide the reader through the sequence of events. Additionally, figure discussion is revised to clarify the labels, ensuring a better understanding of the model structure and logic.

7. “Formalizing the Aspect Threats using Restriction Rules” and “Formalizing the Aspect Mitigations using Restriction Rules” .These two paragraphs need to be rephrased more clearly and concisely, and better organized, to make them more understandable when reading.

**Response:**

The sections titled *“****Formalizing the Aspect Threats using Restriction Rules****”* and *“****Formalizing the Aspect Mitigations using Restriction Rules****”* have been rephrased for improved clarity, conciseness, and organization. In line with the comment we believe that these revisions enhance readability and ensure a clearer understanding of the formalization process.

8. Who is the actor and where is the trigger

**Response:**

Tables 1 and 2 in the manuscript clearly list all the fields used in the Aspect Misuse Case and Aspect Mitigation Case templates, including the actor. It is adopted in accordance with the already published RUCM and RMCM templates, elements such as the actor and other common fields remain valid and applicable within our study. The primary actors are already defined in both RUCM and RMCM and have therefore been retained where appropriate. To directly address this comment and enhance visual clarity, we have updated **Figure 9** to explicitly include the primary actor within the AFMUC template.

Regarding the *trigger* field, we would like to clarify that this concept is not part of the original RUCM or RMCM templates. Given that RMCM forms the foundational basis of our proposed AFMUC approach, we have maintained consistency by not incorporating the trigger field. Its absence in Tables 1 and 2 is therefore intentional and aligned with the RMCM structure.

In line with this comment, the manuscript has been updated to clearly describe the fields adopted and modified from the original RMCM template.

.

9. "**Dependent and independent variables section** “It is better to rephrase this paragraph in a numbered list format for the purpose of organization

**Response:**

The **'Dependent and Independent Variables'** subsection has been revised and reformatted into a numbered list for improved clarity and organization with individual subheading as **“Dependent variable”** and **“independent variable”**

10. formula to calculate the error rate for each restriction rule , is it the authors' own innovation or taken from a source? If it is taken from a source, it must be properly cited.

**Response:**

The formula to calculate the error rate is adapted from an existing source RUCM, where it was originally defined for use case restriction rules cited as [30][39]. In our study, the formula has been modified to suit the context of misuse cases within an aspect-oriented approach. The relevant source has been cited appropriately, and we have also included supporting data that demonstrates the adaptation and modification of these formulas in our context.

To address this comment, the manuscript has been rephrased as a line to provide a clearer explanation regarding the contextual adaptation of the error rate formula. Specifically, the subsection *“****Error Rate Measure”*** under the main section ***“Planning and Design”*** of Experimentation has been revised to enhance understanding. .

11. What are the potential applications that could benefit from the proposed approach.

**Response:**

We have updated the “**discussion**” section with the potential benefit of the proposed approach. Specifically, AFMUC’s modular design helps security analysts clearly specify crosscutting security requirements in complex systems. It also enables security testers to accurately identify attack surfaces and apply targeted mitigations. It is explained in the third paragraph of “**discussion**” section.

12. Add a comparison analysis table between the proposed system and the related works

**Response:**

We have incorporated a structured and detailed comparison in the revised manuscript by introducing three dedicated subsections under the Discussion section:

1. Comparison with existing (mis)use case approaches – supported by Tables 9 and 10
2. Comparison with UML-based approaches – presented in Table 11
3. Comparison with traditional threat modeling approaches – provided in Table 12

Each subsection includes a comprehensive detail and a summary table that presents relevant existing studies. These comparisons highlight the key differences and unique contributions of the proposed AFMUC approach relative to prior works. We believe this structured presentation enhances clarity and meets the intent of the reviewer’s recommendation.

13. Explain system limitation

**Response:**

In response, the manuscript has been updated to include a dedicated “**Limitations**” subsection under the main “**Discussion**” section. This addition provides a balanced reflection on the constraints of the proposed AFMUC approach and outlines scenarios where it may face challenges, thereby offering a more comprehensive and transparent evaluation of the work.

14. Add future works or recommendations

**Response:**

The manuscript has been updated to include a dedicated **“Future Work”** section. This section outlines potential directions for extending the current research, including broader empirical evaluation, application to more complex case studies, and enhancement of the AFMUC framework. These recommendations aim to guide future efforts and strengthen the applicability of the proposed approach.

**CONCLUDING REMARKS**: Again, thank you for giving us the opportunity to strengthen our manuscript with your valuable comments and suggestions. We have worked hard to incorporate your feedback and hope that this revision convinces you to publish our manuscript.

Sincerely,

Corresponding Authors:

**Shafiq ur Rehman**

Kingdom of Saudi Arabia

Email Address: [srehman@imamu.edu.sa](mailto:srehman@imamu.edu.sa)
